# ﻿The first record of *Chironomusnuditarsis* Keyl, 1961 from Sevan Lake (Armenia) confirmed by morphology, karyotype and *COI* gene sequence

**DOI:** 10.3897/compcytogen.18.126130

**Published:** 2024-07-12

**Authors:** Viktor Bolshakov, Alexander Prokin, Elena Ivanova, Ekaterina Movergoz

**Affiliations:** 1 Papanin Institute for Biology of Inland Waters Russian Academy of Sciences, Yaroslavl reg., Nekouz dist., Borok, 152742, Russia Russian Academy of Sciences Borok Russia; 2 Cherepovets State University, Lunacharski 5, Cherepovets 162600, Russia Cherepovets State University Cherepovets Russia

**Keywords:** Barcode, *Chironomusnuditarsis*, *COI*, Diptera, karyotype, Lake Sevan

## Abstract

*Chironomusnuditarsis* Keyl, 1961 is recorded from Sevan Lake for the first time. This species is widespread in Europe, the Caucasus, and Siberia. For species identification, we used a comprehensive approach that included morphological, cytogenetic and molecular genetic analyses. Morphological analysis showed a high similarity with the description. Nine chromosome banding sequences ndtA1, ndtA2, ndtB2, ndtC1, ndtD1, ndtE1, ndtF1, ndtG1, and ndtG2 were found. The banding sequences ndtA1, ndtA2, ndtG1, and ndtG2 are species-specific for *C.nuditarsis* and allow us to accurately distinguish it from the sibling species *Ch.curabilis* Belyanina, Sigareva et Loginova, 1990. Molecular-genetic analysis of the *COI* gene sequences has shown low genetic distances of 0.38–0.95% in the sibling species *Ch.nuditarsis* and *Ch.curabilis* complex and the insufficiency of using a single *COI* as a molecular marker for their separation.

## ﻿Introduction

The study of chironomids from Sevan Lake began in 1936 – 1938, when more than 1,500 midges and 220 larvae and pupae were collected (Pankratova et al. 1980). Initially, only three species from the genus *Chironomus* Meigen, 1803 were recorded from Sevan Lake: *Ch.plumosus* (Linnaeus, 1758), *Ch.tentans* (Fabricius, 1805), and *Ch.thummi* Kief. (now *Ch.riparius* Meigen, 1804) (Fridman 1950; Sharonov 1951; Pankratova et al. 1980). Later, the list was expanded with new species: *Ch.markosjani* Shilova, 1983 (Shilova 1983), which is probably a sibling species to *Ch.annularius* Meigen, 1818 (Bolshakov et al. 2023), inhabiting depths of 10–20 m; *Ch.piger* Strenzke, 1959 and *Ch.dorsalis* Meigen, 1818 (Shilova and Zelentsov 1988); *Ch.bonus* Shilova et Dzhvarsheishvili, 1974 (Kiknadze et al. 2016); *Ch.pilicornis* Fabricius, 1787 (Shcherbina 2016).

In one of the samples from Sevan Lake, we found one *Ch.nuditarsis* Keyl, 1961 larva among other aquatic organisms. Unfortunately, we did not have the opportunity to collect more samples of this species. We regularly suggest to use a comprehensive approach to the species identification of the genus *Chironomus*, which includes morphology, cytogenetics and molecular genetics (Bolshakov and Movergoz 2022; Bolshakov et al. 2022a, 2022b). In this study, we have decided to use a single *Chironomus* larva to obtain a complex of scientific data.

Despite the long history of Sevan Lake *Chironomus* investigations, the list of species is still incomplete, and possibly the fauna is enriched with new species due to changing environmental conditions in the lake, as a result of climate change and human activity, such as water level regulation connected with electricity production in the Hrazdan River cascade of power plants, irrigation of agricultural lands, etc. (Hakobyan and Jenderedjian 2016). In this article, we have added the 8^th^ species *Chironomusnuditarsis* Keyl, 1961 to the list of *Chironomus* of Sevan Lake. Previously, this species has already been recorded for Armenia from the Hrazdan River, Aghrlich (the Artashar Canal) (Petrova et al. 2011) without the karyotype data.

Dr. Fischer has done excellent work on the biology, physiology and genetics of *Ch.nuditarsis* (Rosin and Fischer 1966; Fischer 1969, 1974; Fischer and Rosin 1969; Fischer and Tichy 1980; Adamek 2020); thanks to the study of the biology of *Ch.nuditarsis*, it became possible to maintain a laboratory culture of this species (Fischer 1969). A detailed morphological description of the imago was later performed by Klotzli (Klotzli 1974).

The studied species *Ch.nuditarsis* has a wide Palearctic range; therefore it is a convenient object for studying the chromosomal divergence of populations and species (Polukonova et al. 2005a, 2005b; Kiknadze et al. 2006; Jabłońska-Barna and Michailova 2009; Kiknadze et al. 2016; Karmokov 2020). Chromosome maps of *Ch.nuditarsis* have been described for larvae collected in Germany by Keyl (Keyl 1961). In Caucasian populations, inversion polymorphism was observed for the majority of chromosomal arms, except for C and E (Karmokov 2020).

P. Michailova (Michailova 1989) found two karyological races in Bulgarian populations of *Ch.nuditarsis*, one with small centromeric bands and the other with large centromeric bands (Michailova 1989). Later it was found that the second karyological race, with large centromeric bands, corresponds to the species *Ch.curabilis* Belyanina, Sigareva et Loginova, 1990, which was originally described by morphological characteristics and the karyotype was mapped later (Beljanina et al. 1990) according to the Maximova system (Maximova 1976). Some authors argued that *Ch.nuditarsis* did not belong to any group according to morphological characters, although its position on the tree constructed according to cytogenetic data indicated its high affinity to the *Ch.plumosus* group (Kiknadze et al. 2004). Thus, *Ch.nuditarsis* and *Ch.curabilis* were placed in the *Ch.nuditarsis* group of sibling species (Polukonova et al. 2005b). However, the results of molecular genetic analysis further supported the suggestion that both species belong to the *Ch.plumosus* group (Djomin 2011; Karmokov 2019; Bolshakov and Movergoz 2022; Bolshakov et al. 2022a) so both species are now considered members of the *Ch.plumosus* group.

The *COI* gene sequences for *Ch.nuditarsis* are known for only two regions, Germany and the United Kingdom; the species was identified by larval morphology and polytene chromosomes (Pfenninger et al. 2007), and by using only eDNA (Bista et al. 2017), respectively. Unfortunately, the length of the *COI* gene sequences of *Ch.nuditarsis* corresponding to Germany is only 416 nucleotides.

Traditionally, many samples of the *COI* gene sequence used for phylogenetic analysis were obtained using only one identification method based on imago or larva morphology, which can often lead to a misidentification, thus working with *COI* gene of chironomids requires an integrated approach (DeSalle et al. 2005; Ekrem et al. 2007; Kondo et al. 2016; Bolshakov and Movergoz 2022).

Occasionally, samples are received from locations that are difficult to access or infrequently visited, and there is no opportunity to return to collect the material. Consequently, every sample is of significant value, and we endeavor to obtain as much information as possible about each *Chironomus* larva. In this study, we demonstrate that even a single individual of *Ch.nuditarsis* from Lake Sevan can be studied using a comprehensive approach that includes morphological, cytogenetic, and molecular genetic methods.

## ﻿Material and methods

One IV instar larva was found in Sevan Lake (Artanish Bay), Gegharkunik Province, Armenia (among other macroinvertebrates in the macrozoobenthos sample) on October 10, 2019. Coordinates – 40.462450, 45.355983. The depth at the sampling site was 1.3 m, and the bottom sediments were black silted gravel.

The larva was fixed in 96% ethanol. For morphological analysis, the head capsule of larva was mounted on a slide in the Fora-Berlese solution, the morphological terminology proposed by Sæther (Sæther 1980) was used. The age was determined by the standard method (Ilyinskaya 1983). The salivary glands were extracted through an incision (1–3 body segments) with thin preparation needles. Karyotype analysis was performed using the ethanol-orcein method (Dyomin 1989). The specimens were analyzed with a light microscope (Micromed-6 LOMO, St-. Petersburg, Russia) with an objective of ×100 and a digital camera (ToupCam 5.1., ToupTek Photonics, Hangzhou, China), and with a light stereomicroscope (Olympus CX43 with digital camera DP23, Olympus, Tokyo, Japan). For identification of chromosome banding sequences previously published cytomaps were used (Kiknadze et al. 2006, 2016; Karmokov 2020). Arms A, E and F were mapped in the system of Keyl (Keyl 1962), and arms C and D – in the system of Dévai et al. (Dévai et al. 1989). The morphological preparation of the head capsule and cytological preparation are deposited in the collection of Papanin Institute for Biology of Inland Waters Russian Academy of Sciences, Russia, Borok (IBIW).

DNA extraction was performed by the “M-sorb-OOM” (Sintol, Moscow) kit with magnet particles according to manufacturer’s protocol. For amplification of *COI* (cytochrome oxidase subunit I) primers LCO1490 (5’-GGTCAACAAATCATAAAGATATTGG-3’) and HCO2198 (5’-TAAACTTCAGGGTGACCAAAAAATCA -3’) were used (Eurogen, Moscow) (Folmer et al. 1994). Amplification reaction was carried out in 25 μl reaction mixture (1× buffer, 1.5 μM MgCl2, 0.5 mM of each primer, 0.2 μM dNTP of each nucleotide, 17.55 μL deionized water, 1 μL template DNA, 1 unit Taq-polymerase (Evrogen, Moscow). PCR was performed at 94 °C (3 min), followed by 30 cycles at 94 °C (15 s), 50 °C (45 s), 72 °C (60 s) and a final at 72 °C (8 min). PCR products were visualized in1% agarose gel and purified with ethanol and ammonium acetate (3 M). Both strands were sequenced on an Applied Biosystems 3500 DNA sequencer (Thermo Scientific, USA) following the manufacturer’s instructions.

For alignment of *COI* nucleotide sequences we used MUSCLE in the MEGA6 software (Tamura et al. 2013). Pairwise genetic distances were calculated in the MEGA6 software using Kimura 2-parameter (K2P) with codon position preferences: 1^st^, 2^nd^, 3^rd^ and noncoding sites. The program MrBayes v.3.2.6 was used for the Bayesian analysis (Ronquist and Huelsenbeck 2003; Ronquist et al. 2012) with previously suggested settings (Karmokov 2019; Bolshakov et al. 2022a), for 1 000 000 iterations and 1000 iterations of burn-in, nst = 6 (GTR + I + G). The phylogenetic trees resulting from Bayesian inference analyses were visualized and edited using FigTree v.1.4.3 (http://tree.bio.ed.ac.uk/software/figtree/). For the estimation of the number of haplotypes we used DNA SP v.6 software (Librado and Rozas 2009), and to create a network of haplotypes we used PopArt 1.7 software, with the Median Joining algorithm (Leigh and Bryant 2015).

In addition, thirty one *COI* gene sequences of the genus *Chironomus* from GenBank were analyzed. Accession numbers of used sequences in GenBank: *Ch.acutiventris* Wülker, Ryser et Scholl, 1983 (AF192200), *Ch.annularius* Meigen, 1818 (AF192189), *Ch.anthracinus* Zetterstedt, 1860 (KF278222), *Ch.balatonicus* Dévai, Wülker et Scholl, 1983 (JN016826), *Ch.bernensis* Klötzli, 1973 (AF192188), *Ch.borokensis* Kerkis, Filippova, Shobanov, Gunderina et Kiknadze, 1988 (AB740261), *Ch.cingulatus* Meigen, 1830 (AF192191), *Ch.commutatus* Keyl, 1960 (AF192187), *Ch.curabilis* Belyanina, Sigareva et Loginova, 1990 (MT535221, JN016811, KX118693), *Ch.entis* Shobanov, 1989 (AF192195), *Ch.heteropilicornis* Wülker, 1996 (MK795770), *Ch.luridus* Strenzke, 1959 (AF192203), *Ch.maturus* Johannsen, 1908 (DQ648204), *Ch.melanescens* Keyl, 1961 (MG145351), *Ch.melanotus* Keyl, 1961 (OL546775), *Ch.novosibiricus* Kiknadze, Siirin et Kerkis, 1993 (AF192197), *Ch.nuditarsis* Keyl, 1961 (KY225345), *Ch.pallidivittatus* Malloch, 1915 (AF110164), *Ch.piger* Strenzke, 1959 (AF192202), *Ch.pilicornis* Fabricius, 1787 (HM860166), *Ch.plumosus* Linnaeus, 1758 (AB740263), *Ch.riparius* Meigen, 1804 (KR756187), *Ch.sokolovae* Istomina, Kiknadze et Siirin, 1999 (MW471100), *Ch.sororius* Wülker, 1973 (MZ324811), *Ch.tentans* Fabricius, 1805 (AF110157), *Ch.tuvanicus* Kiknadze, Siirin et Wülker, 1993 (AF192196), *Ch.usenicus* Loginova et Belyanina, 1994 (JN016806), *Ch.whitseli* Sublette et Sublette, 1974 (KR683438). Species *Drosophilamelanogaster* Meigen, 1830, Drosophilidae (HQ551913) was used as an out-group in phylogenetic analysis.

In order not to miss the details, we used all 25 available *COI* gene sequences of *Ch.nuditarsis* and *Ch.curabilis* from the GenBank database, including short ones (416 bp). *Ch.nuditarsis*: Germany (DQ910577, DQ910575, DQ910573, DQ910568, DQ910574, DQ910576, DQ910569, DQ910570, DQ910567, DQ910579, DQ910578, DQ910572, DQ910571). *Ch.curabilis*: Montenegro (MT535377, MT535005, MT534682, MT534976), Germany (OP927609, OP927503, OP927448, OP927684, OP927470, OP927434) and Russia, Saratov reg. (JN016810, JN016812).

### ﻿Study area

Sevan Lake is the largest freshwater lake in Armenia and the Caucasus region; it is located in the northern part of the Armenian Volcanic Highland at an altitude of approximately 1,900 meters above sea level, with a surface of 1278.04 km^2^ (Hakobyan and Jenderedjian 2016; Krylov 2016; Jenderedjian et al. 2019).

We suggest that in the future, knowledge of the vegetation at chironomid larvae collection sites may help to reveal the relationship with species richness. The study of the aquatic vegetation of Sevan Lake is conducted regularly; *Cladophoraglomerata* (L.) Kütz., *Myriophyllumspicatum* L. were registered in sampling site. The composition of the aquatic core of the flora also includes: *Vaucheriadichotoma* (L.) Mart., *Drepanocladusaduncus* (Hedw.) Warnst., *Butomusumbellatus* L., *Potamogetonpectinatus* L., *P.perfoliatus* L., *Ceratophyllumdemersum* L., *Lemnagibba* L., *L.trisulca* L., Salix elbursensis Boiss, *Schoenoplectustabernaemontani* (C.C.Gmel.) Palla, *Typhaangustifolia* L., *Butomusumbellatus* L., *Phragmitesaustralis* (Cav.) Trin. ex Steud. (Arnoldi 1929; Barsegyan 1975; Tsaturyan et al. 1985; Bobrov 2016).

## ﻿Results

### ﻿Morphological characters of *Ch.nuditarsis* from Sevan Lake

**Figure 1.**
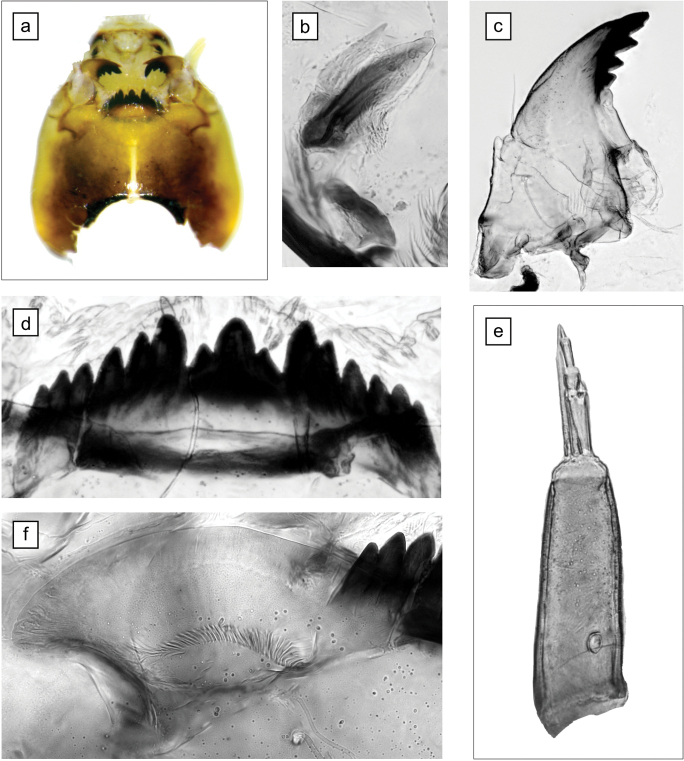
Larva morphology of *Ch.nuditarsis* from Sevan Lake, Armenia **a** head capsule, ventrally **b** premandible **c** mandible **d** mentum **e** antenna **f** ventromental plate. Scale bar: 100 µm.

The morphological characteristics of the 4^th^ instar larva are shown in Fig. 1. The body length was about 15 mm. The length of the ventral tubules exceeds the length of the posterior parapods. The head capsule was yellow or light brown (Fig. 1a). The gular spot has blurred borders. The exterior tooth of the premandible is significantly narrower (3 times) than the inner tooth (Fig. 1b). All four teeth of the mandible are dark brown (Fig. 1c). The mentum is black-brown; the fourth tooth of the mentum is not lower than the neighboring teeth (Fig. 1d). The basal segment of the antenna is cone-shaped; the antenna blade is extended above the fifth segment (Fig. 1e). Ventromental plates have a flat front edge and without a wrinkly surface (Fig. 1f).

The measurement results: length of the first antennal segment (L1) - 141 µm, length of the second segment (L2) - 37 µm, width of the first segment (W1) - 49 µm; the distance of the ring organ from the base of the first antenna segment - 43 µm; mental size (MS), the distance between the first lateral teeth - 91 µm; number of epipharyngeal teeth – 14.

### ﻿Karyotype of *Chironomusnuditarsis* from Sevan Lake

**Figure 2.**
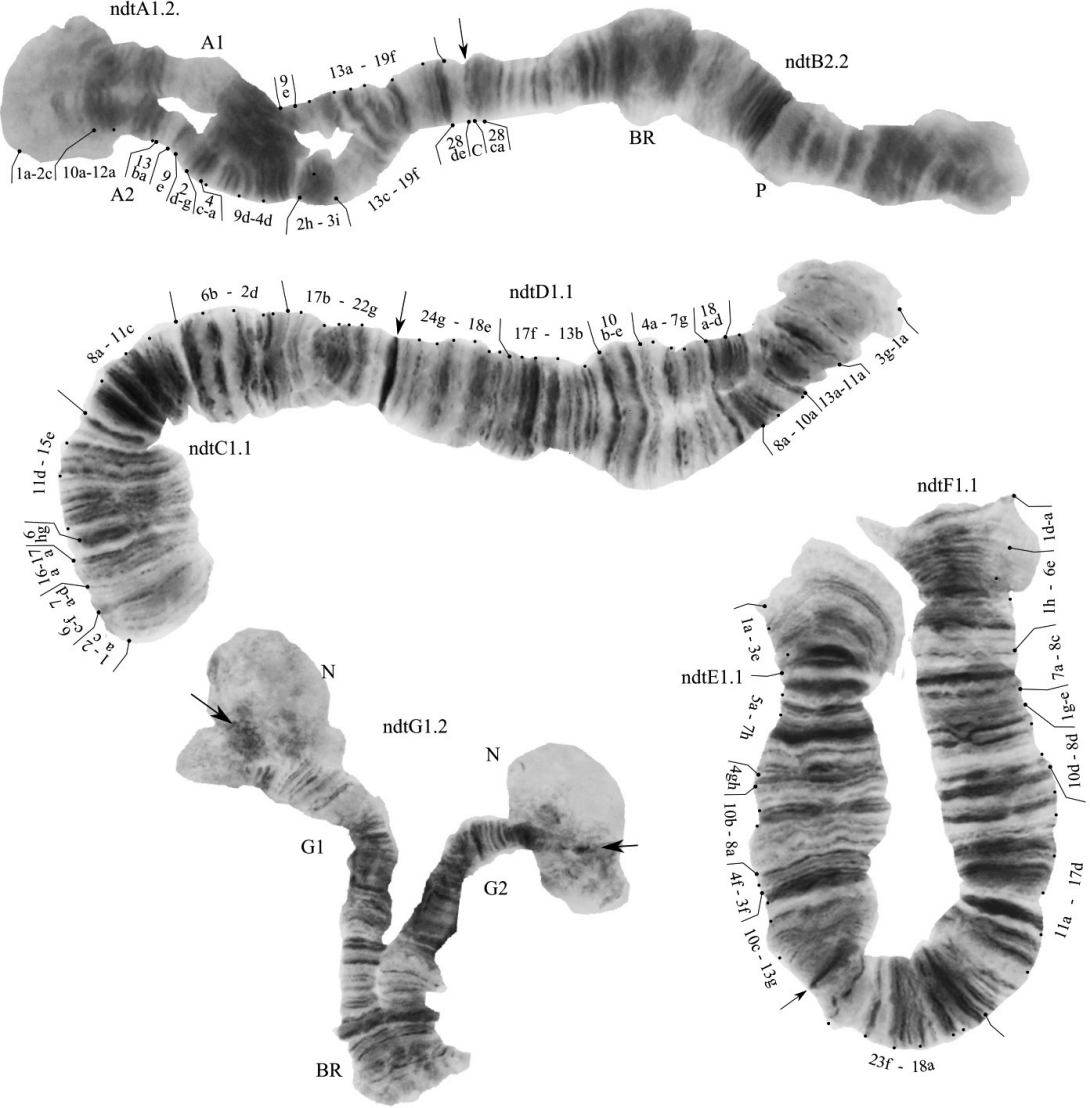
Karyotype of *Ch.nuditarsis* from Sevan Lake, Armenia. Arrows indicate centromeric bands; ndtA1.2, ndtB2.2., etc., genotypic combinations of banding sequences of chromosome arms; BR, Balbiani rings; N, nucleolus.

The species has a 2n = 8 set of chromosomes. The chromosome arm combination is AB CD EF G (“thummi” cytocomplex). The chromosomes AB and CD are metacentric, EF is submetacentric, short G is telocentric. Balbiani rings are located in arms B and G, nucleolus is located in arm G (Fig. 2).

One zygotic combination was found: ndtA1.2.B2.2.C1.1.D1.1.E1.1.F1.1.G1.2. All nine banding sequences in our study were previously described from different populations (Michailova et al. 2002; Polukonova 2005b; Kiknadze et al. 2006, 2016; Karmokov 2020).

**Arm A.** Two banding sequences: ndtA1 1a-2c 10a-12c 3i-2h 4d-9d 4a-c 2g-d 9e 13a-19f [28de] C. in heterozygous state with ndtA2 1a-2c 10a-12a 13ba 9e 2d-g 4c-a 9d-4d 2h-3i 12cb 13c-19f [28de] C.

**Arm B.** One banding sequence: ndtB2, not mapped.

**Arm C** One banding sequence: ndtC1 1a-2c 6c-f 7a-d 16a-17a 6hg 11d-15e 8a-11c 6b-2d 17b-22g C.

**Arm D.** One banding sequence: ndtD1 1a-3g 11a-13a 10a-8a 18d-a 7g-4a 10e-b 13b-17f 18e-24g C.

**Arm E.** One banding sequence: ndtE1 1a-3e 5a-7h 4gh 10b-8a 4f-3f 10c-13g C.

**Arm F** One banding sequence: ndtF1 1a-d 6e-1h 8c-7a 1e-g 8d-10d 17d-11a 18a-23f C.

**Arm G.** Two banding sequences: ndtG1, not mapped and ndtG2,not mapped.

*Ch.nuditarsis* has many banding sequences similar to *Ch.curabilis*; in *Ch.nuditarsis* from Sevan Lake we have found four banding sequences which are considered specific for *Ch.nuditarsis*: ndtA1, ndtA2, ndtG1 and ndtG2 (Polukonova et al. 2005b).

### ﻿COI gene sequences and phylogenetic analysis of *Ch.nuditarsis* from Sevan Lake

The obtained *COI* gene sequence of *Ch.nuditarsis* from Sevan Lake was deposited in GenBank with the accession number - OR652398, length - 658 bp. Percentage of nucleotides A: 26; T: 37; G: 17; C: 20.

We found one *COI* gene sequence length of 608 bp in the GenBank that belongs to *Ch.nuditarsis* (KY225345), other sequences had a length of 416 bp, and this decreased the accuracy of the analysis. The minimum genetic distances were between *COI* gene sequences *Ch.nuditarsis* (OR652398) from Sevan Lake and *Ch.curabilis* (KX118693) from Iran – 0.49%, and *Ch.curabilis* (JN016811) from Saratov reg. (Russia) – 0.73% (Table 1). The genetic distance between *COI* gene sequences of *Ch.nuditarsis* (OR652398) from Sevan Lake and *Ch.nuditarsis* (KY225345) from the United Kingdom was 0.98%. Another low genetic distance of 1.23% was found between *Ch.nuditarsis* (OR652398) from Sevan Lake and *Ch.curabilis* (MT535221) from Skadar Lake in Montenegro. Almost all the estimated distances between sequences of *Ch.nuditarsis* and *Ch.curabilis* were less than the accepted interspecific threshold value of 3% (Ekrem et al. 2007; Proulx et al. 2013; Kondo et al. 2016), the only exception is the distance of 3.75% between *Ch.nuditarsis* (DQ910574) from Germany and *Ch.curabilis* (JN016812) from Saratov region (Russia). The distances between sequences of *Ch.nuditarsis* and other species from the *Ch.plumosus* group varied from 4.01 to 8.54% (Table 1), exceeding the interspecific threshold value of 3% (Ekrem et al. 2007; Proulx et al. 2013; Kondo et al. 2016).

**Table 1.** The pairwise genetic distances (Kimura-2p) between *COI* gene sequences of *Chironomus* species.

No.	1	2	3	4	5	6	7	8	9	10	11
	*Ch.nuditarsis* (OR652398) Sevan Lake	*Ch.nuditarsis* (KY225345) United Kingdom	*Ch.nuditarsis* (DQ910574) Germany	*Ch.curabilis* (KX118693) Iran	*Ch.curabilis* (JN016811) Saratov reg. Russia	*Ch.curabilis* (MT535221) Montenegro	*Ch.curabilis* (JN016812) Saratov reg. Russia	*Ch.usenicus* (JN016806) Russia	*Ch.plumosus* (AB740263) Russia	*Ch.entis* (AF192195) Russia	*Ch.borokensis* (AB740261) Russia
1											
2	0.98										
3	2.23	2.23									
4	0.49	0.98	2.22								
5	0.73	1.23	2.99	1.23							
6	1.23	0.73	2.48	1.23	1.48						
7	1.47	1.97	3.75	1.97	0.73	2.22					
8	4.27	4.01	5.84	4.53	4.26	3.75	5.04				
9	4,53	4,26	6,10	4,78	4,52	4,52	5,30	0,73			
10	6,37	6,10	8,54	6,36	6,09	6,36	6,89	5,06	5,85		
11	5,86	5,59	7,47	6,12	5,85	5,85	6,65	3,00	3,25	7,50	

**Figure 3.**
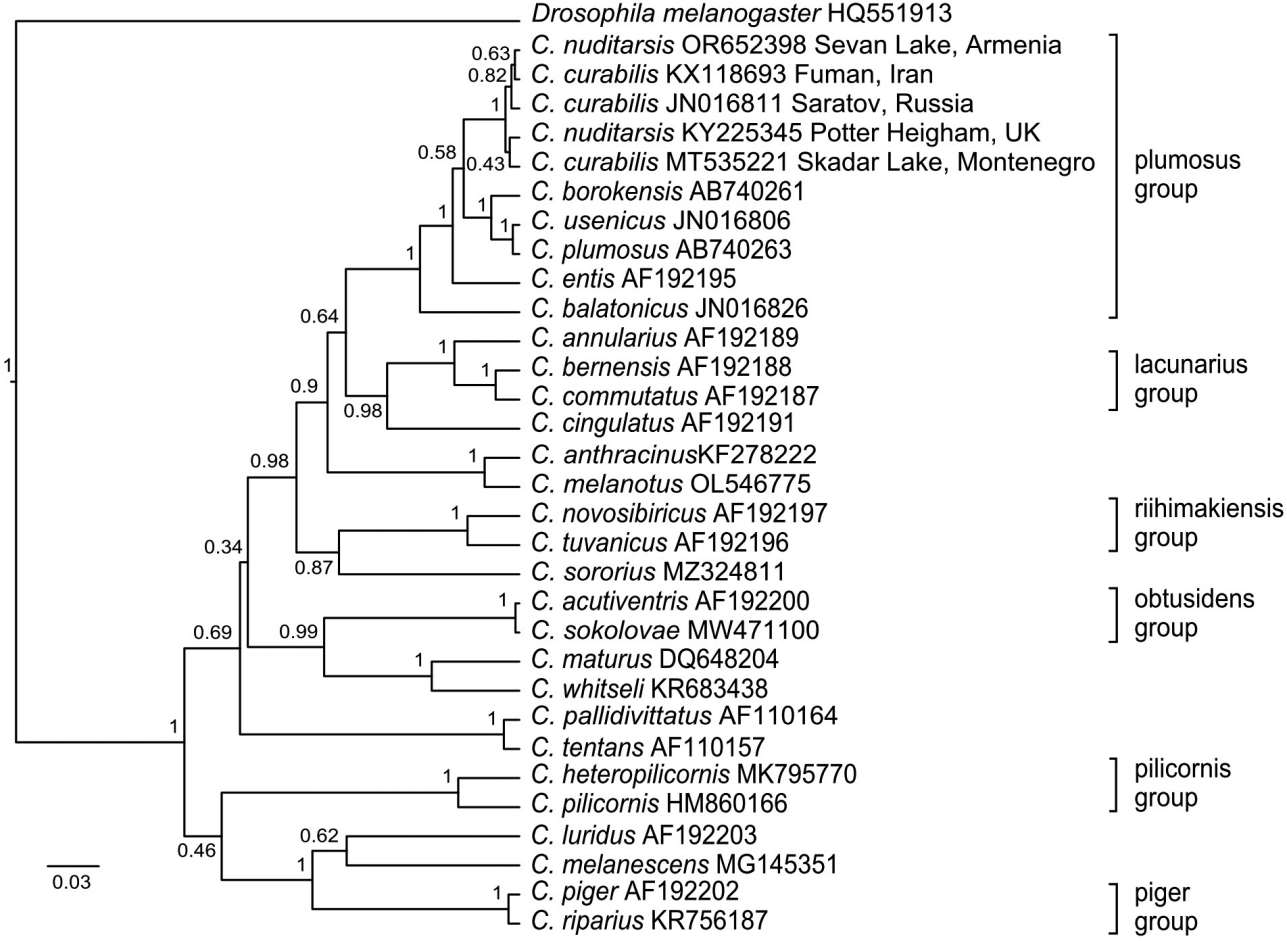
Bayesian tree of the analyzed samples of *Chironomus* spp., inferred from *COI* gene sequences. Species name, GenBank accession numbers and group name are shown to the right of the branches. Support p-values are given if they exceed 0.3.

*COI* gene sequences of *Ch.nuditarsis* (OR652398) from Sevan Lake form a single cluster with *Ch.curabilis* from Iran (KX118693), Russia (JN016811) and Montenegro (MT535221), and *Ch.nuditarsis* (KY225345) from the United Kingdom (Fig. 3). It can be assumed that only the samples of *Ch.curabilis* (JN016811, JN016812) from Saratov region (Russia) were identified by cytogenetics (Polukonova et al. 2009), the others by morphology (Chimeno et al. 2023), or only by *COI* gene sequence (Bista et al. 2017). All short sequences of *Ch.nuditarsis* and *Ch.curabilis* were combined into one large cluster; we do not consider it fitting to give this figure here. All short sequences of *Ch.nuditarsis* and *Ch.curabilis* were used to create a haplotype network.

**Figure 4.**
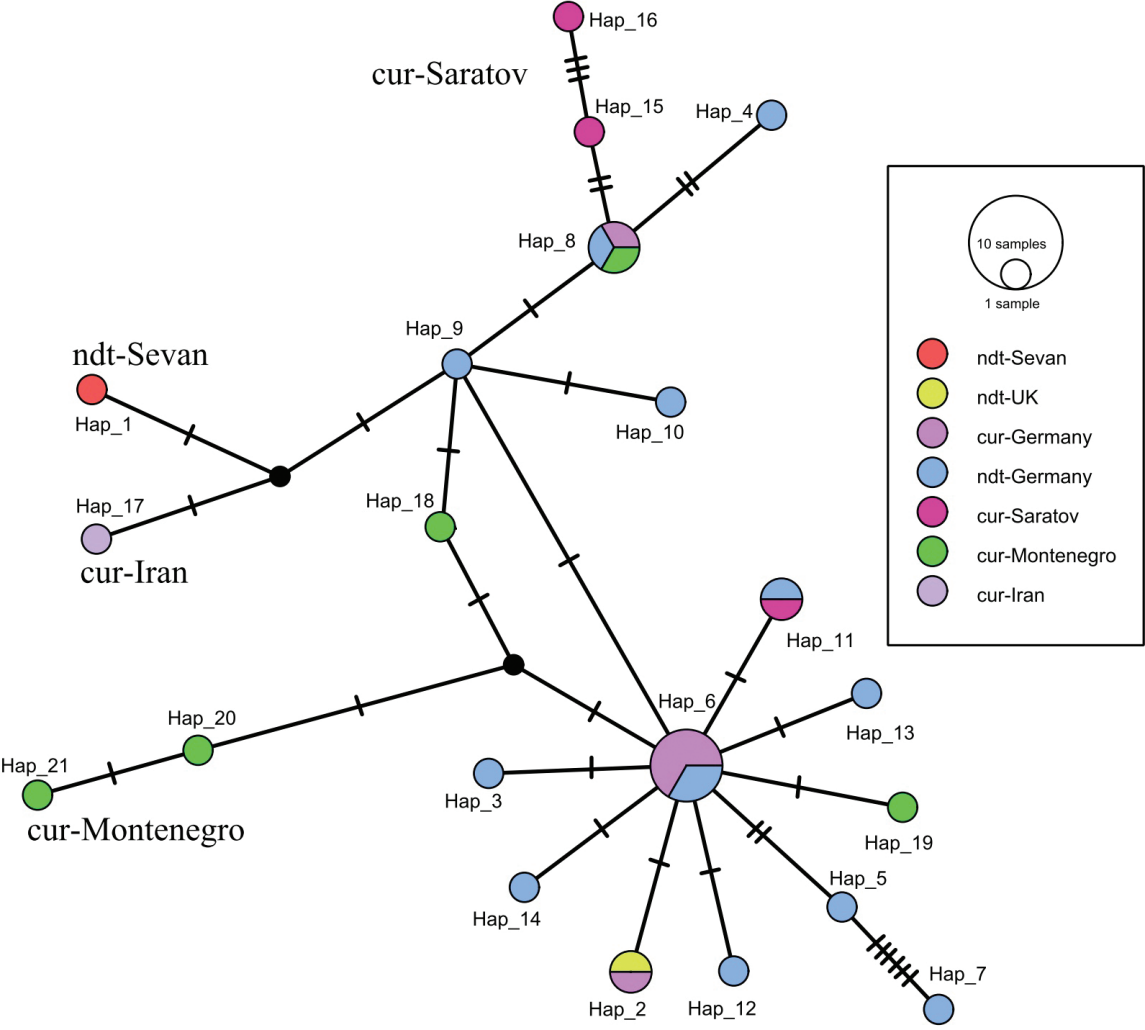
Median Joining Network showing phylogenetic relationships within *Ch.nuditarsis* and *Ch.curabilis* species. Each bar represents a single mutational change. The diameter of the circles is proportional to the number of individuals in each haplotype sampled (see open circles with numbers). Black dots represent hypothetical intermediate haplotypes. Sevan, Saratov, etc. – names of localities.

The resulting haplotype network of *Ch.nuditarsis* and *Ch.curabilis* has a quite complex structure and consists of 21 haplotypes (Fig. 4). *COI* gene sequences of both species have the same haplotypes. Haplotype 2 consists of *Ch.nuditarsis* (KY225345) from the United Kingdom and *Ch.curabilis* (OP927684) from Germany. Haplotype 6 includes *COI* gene sequences of *Ch.nuditarsis* (DQ910568, DQ910570) and *Ch.curabilis* (OP927609, OP927448, OP927470, OP927434) from Germany. Haplotype 8 includes sequences of *Ch.nuditarsis* (DQ910576) and *Ch.curabilis* (OP927503) from Germany, and *Ch.curabilis* (MT535377) from Montenegro. The maximum number of mutation steps (20) was found between *Ch.curabilis* (JN016812) from Saratov region, Russia, and *Ch.nuditarsis* (DQ910574) from Germany. The haplotype of *Ch.nuditarsis* (OR652398) from Sevan Lake is separated from *Ch.nuditarsis* (DQ910569) from Germany and *Ch.curabilis* (KX118693) from Iran by three mutation steps.

## ﻿Discussion

Hydrobiological and entomological investigations, including studies of chironomids, are performed regularly in Lake Sevan (Pankratova et al. 1980; Shilova 1983; Shilova and Zelentsov 1988; Shcherbina and Zelentzov 2011; Hakobyan and Jenderedjian 2016; Shcherbina 2016). As a result of that long-term study, a total of 8 species of the genus *Chironomus* are known today from the lake, but some of them still need confirmation using a comprehensive approach.

In Artanish Bay, the locality of our material of *Ch.nuditarsis*, previously only *Ch.tentans* was recorded (Shilova and Zelentsov 1988). We assume that species *Ch.nuditarsis* has not been found for a long time previously due to the high diversity of habitat conditions in Sevan Lake (Pavlov et al. 2010; Hakobyan and Jenderedjian 2016; Krylov 2016; Jenderedjian et al. 2019), and the possible changing of the fauna. Incredibly, chironomids were found at a depth of up to 63 meters in 2015, because of the relatively high concentration of dissolved oxygen in this period in the profundal zone (Hakobyan and Jenderedjian 2016).

During the course of preparing this article, we encountered some confusion regarding the author of the name for the species in question. The author of the species, at the first mention (Keyl 1961), attributes name authorship to Strenzke (Strenzke 1959), who in reality did not use such a name in the cited publication. In addition, Dr. Fischer’s articles indicate the species name as *Ch.nuditarsis* Str., yet the year is not specified (Fischer 1969, 1974; Fischer and Rosin 1969). The publication that provides a detailed description of the imago indicates *Ch.nuditarsis* Keyl, 1961 (Klotzli 1974). In a comprehensive article on the morphological characteristics of *Chironomus* larvae, this species is listed as *Ch.nuditarsis* Keyl, 1961 (Webb and Scholl 1985). In Polukonova (Polukonova 2005a) two alternative names are used, where different years of description are indicated *Ch.nuditarsis* Keyl, 1962 and *Ch.nuditarsis* Str. (Keyl, 1961) (Polukonova 2005a). Later, in an article by Jabłońska-Barna and Michailova, the species was named *Ch.nuditarsis* Strenzke, 1959 (Jabłońska-Barna and Michailova 2009). In recent works by Kiknadze et al. (Kiknadze et al. 2016) and Karmokov (Karmokov 2020) the species is named *Ch.nuditarsis* Keyl, 1961. To sum up, the work of Keyl (Keyl 1961) should be accepted as the first mention and original description of the species.

The morphological characteristics of the larva we found correspond to the description of *Ch.nuditarsis* (Webb and Scholl 1985; Polukonova 2005a) and differs from the sibling species of *Ch.curabilis*. The head capsule of *Ch.nuditarsis* is darker (dark yellow or brown), the gular spot has blurred borders, in *Ch.curabilis* the head capsule is yellow and has a clear gular spot (Webb and Scholl 1985; Polukonova 2005a). In *Ch.nuditarsis*, the basal segment of the antenna is cone-shaped; the antenna blade is extended beyond the fifth segment; this is well illustrated in Fig. 1e. In *Ch.curabilis*, the basal segment of the antenna is cylindrical; the antenna blade reaches the base of the fifth segment (Webb and Scholl 1985; Polukonova 2005a). The measurements of the most significant morphological characteristics also demonstrated a match with the description of *Ch.nuditarsis* (Webb and Scholl 1985; Polukonova 2005a).

The cytogenetic analysis of *Ch.nuditarsis* in Caucasian populations demonstrated a high diversity of chromosome banding sequences (Kiknadze et al. 2006; Karmokov 2020), explained by the authors as a result of high diversity of habitat conditions. Some chromosome banding sequences registered in Sevan Lake are rare among Caucasian populations. Heterozygote ndtA1.2 in the populations of the Central Caucasus was found only in two localities of Kabardino-Balkaria, with the maximum frequency of occurrence of 1.1–1.8%, but it is common in the populations of the Northwestern Caucasus and Europe, with the frequency of occurrence of about 30–40% (Karmokov 2020). In former studies of *Ch.nuditarsis* in Caucasian populations the ndtA2 banding sequence was not observed at all (Polukonova and Karmokov 2013). Homozygote ndtB2.2 is common for Siberian populations and occurs in all individuals; it is rare in Central Caucasus and European populations and more common in the northwest and east Caucasus (Kiknadze et al. 2006; Karmokov 2020). Heterozygote ndtG1.2 is common in European and Caucasian populations, but not in Siberian populations (Kiknadze et al. 2006; Polukonova and Karmokov 2013; Karmokov 2020). It is noted that the frequency of occurrence of zygotic combination ndtG1.2 may depend on the altitude of the habitat, it is more common in the plains, but rare in the highlands (Polukonova and Karmokov 2013). In addition, the zygotic combinations ndtB2.2 and ndtG1.2 do not follow the Hardy-Weinberg expectation in some Caucasian populations (Karmokov 2020). Based on the obtained data, we can assume that the combination of chromosome banding sequences of the Sevan Lake larva might be more similar to the northwest Caucasian population.

Previously, the presence of two karyological races was noted in Bulgarian populations of *Ch.nuditarsis*, differing in the size of the centromeric region (Michailova 1989). Later, the karyoform with large centromeric bands was associated with the species *Ch.curabilis* (Polukonova et al. 2005b). As noted earlier, karyoforms with large centromeric bands are not found in the Caucasian populations (Karmokov 2020), and the larvae we found in Sevan Lake also has thin centromeric bands. These two species, *Ch.nuditarsis* and *Ch.curabilis*, have many similar chromosome banding sequences (Polukonova et al. 2005b), which is also characteristic of many species of the genus *Chironomus* (Kiknadze et al. 2006, 2008, 2016). It is known that banding sequences ndtA1, ndtA2, ndtG1, and ndtG2 are specific for *Ch.nuditarsis* (Polukonova et al. 2005b), which confirms the accuracy of the identification of the species in this publication.

The estimated genetic distances between *COI* gene sequences of *Ch.nuditarsis* and *Ch.curabilis* were less than the accepted 3% threshold (Ekrem et al. 2007; Proulx et al. 2013; Kondo et al. 2016), only in one case, *Ch.nuditarsis* (DQ910574) from Germany and *Ch.curabilis* (JN016812) from Saratov region (Russia), the distance was 3.75% (Table 1).

To make sure that the species is correctly identified, we studied the publications with available data on the locality and methods for species identification. In the case of *Ch.curabilis* (KX118693) from sediments of Anzali Wetland, Iran, the species was probably identified by the morphology of the larva as the authors usually indicate that they used genus identification without the need for routine mounting of larvae (Salehzadeh et al. 2019). Samples of *Ch.nuditarsis* (DQ910569 – DQ910579) collected on the Rhine River plain in southwestern Germany were identified cytogenetically (Pfenninger et al. 2007). One sample of *Ch.nuditarsis* (KY225345) was collected as part of eDNA, from the water bodies of Potter Heigham, Great Yarmouth, United Kingdom, and was identified only by *COI* gene sequence (Bista et al. 2017). Samples of *Ch.curabilis* (with prefix OP927…) that were collected in Dark-Sky Reserve within the Westhavelland Nature Park in the Berlin-Brandenburg Metropolitan Region, Germany, were identified by morphology of imago (Chimeno et al. 2023). Samples of *Ch.curabilis* (with prefix MT535…) that were collected from the Dark-Sky Reserve within the Westhavelland Nature Park in the Berlin-Brandenburg Metropolitan Region, Germany, were identified by imago morphology (Gadawski et al. 2022). Samples of *Ch.curabilis* (with prefix JN016…) from the Saratov region, Russia, were identified by cytogenetics (Polukonova et al. 2009). As we can see, the species identification in most samples was performed by imago morphology and cytogenetically, and we can consider this data relatively reliable, therefore, the accuracy of the results may be affected by the length of the obtained nucleotide sequences.

On the Bayesian tree, the *COI* gene sequences of *Ch.nuditarsis* and *Ch.curabilis* combined into one cluster (Fig. 3), which combines with the *Ch.plumosus* group, and it matches the previous data (Kiknadze et al. 2004; Pfenninger et al. 2007; Djomin 2011). Some authors suggest to include the species in the *Ch.plumosus* group (Djomin 2011). The group of sibling-species has an artificial character, where species with similar cytogenetic characteristics and morphological features are combined, often without clear criteria (Shobanov 2000). Due to the development of techniques, we believe that it is necessary to add molecular genetic criteria for sibling species separation together with morphology and cytogenetics.

Further calculations only with additional sequences of *Ch.nuditarsis* and *Ch.curabilis* showed that there was no association within the cluster either by species name or by locality. Within a network of haplotypes based on the *COI* gene sequences of both species *Ch.nuditarsis* and *Ch.curabilis* (Fig. 4), we can see the same situation as in the Bayesian tree: sequences are combined into common haplotypes. This is visible in haplotype 6, which contains sequences from Germany of both species. The haplotype of *Ch.nuditarsis* from Sevan Lake differs from the *Ch.curabilis* from Iran and *Ch.nuditarsis* from Germany by three mutational steps. The highest number of mutation steps, 20, was found between *Ch.nuditarsis* (DQ910574) from Germany and *Ch.curabilis* (JN016812) from Saratov reg. (Russia), with the genetic distance of 3.75%, and only in this case species distinguished as separate. At this stage of the study, it is clear that populations from Europe have the greatest diversity, and other haplotypes diverge from them. Unfortunately, we do not have the *COI* gene sequences of samples from Siberia, and we cannot follow the changes from West to East like with chromosomal polymorphism (Kiknadze et al. 2006; Petrova et al. 2013). Although such trends are already being observed, populations from Saratov (Russia), Iran, Sevan Lake (Armenia), and Skadar Lake (Montenegro) are beginning to form separate haplotypes.

This situation indicates a close relationship between two sibling species and the insufficiency of using single *COI* gene as a molecular marker for their separation in the case studied of *Ch.nuditarsis* and *Ch.curabilis*. Previously performed investigations of the diversity of *COI*, *gb2b* gene sequences, and the possibility of their use in species delimitation indicate that the calculated threshold cannot be used to separate all *Chironomus* species (Proulx et al. 2013). Another explanation is interspecific hybridization and horizontal transfer of mitochondrial genes with fixation in one of the initial species in a population (Guryev and Blinov 2002; Polukonova et al. 2009; Karmokov 2019; Bolshakov et al. 2021). Previously, we confirmed the existence of hybrids even between species from different cytocomplexes (Bolshakov et al. 2022b).

Chironomid larvae are an important component of aquatic ecosystems and a model object for ecological and hydrobiological studies, as well as a convenient object for cytogenetics (Keyl 1962; Kiknadze et al. 2016). Recently, the study of chironomids phylogenetic relationships has increasingly used the analysis of mitochondrial genes (Li et al. 2022). New knowledge allows for comprehensive monitoring of the ecological state of the environment. According to the changes in environmental conditions caused by global warming and human activities, it is necessary to continue a long-term study of such unique and regionally important reservoirs as Sevan Lake.

Using the case of *Ch.nuditarsis*, we have shown that even a single larva can be subjected to a comprehensive examination, including morphological, cytogenetic and molecular genetic analysis, and a lot of interesting information can be obtained. Do not ignore even one larva.
